# An approach for proteins and their encoding genes synonyms integration based on protein ontology

**DOI:** 10.1186/s12859-023-05464-0

**Published:** 2023-09-12

**Authors:** Xiaohong Wang, Xiaoli Jing, Fangkun Dou, Haowei Cao

**Affiliations:** 1 Teaching Affairs Department, Shandong Foreign Trade Vocational College, Qingdao, 266100 China; 2 Network and Information Center, Qingdao Marine Science and Technology Center, Qingdao, 266237 China; 3 Oceanographic Data Center, Institute of Oceanology, Chinese Academy of Sciences, Qingdao, 266071 China; 4 Key Laboratory of Computing Power Network and Information Security, Ministry of Education, Shandong Computer Science Center, Qilu University of Technology (Shandong Academy of Sciences), Jinan, 250000 China; 5 Shandong Provincial Key Laboratory of Computer Networks, Shandong Fundamental Research Center for Computer Science, Jinan, 250000, China

**Keywords:** Ontology, Knowledge graph, Data integration, Synonyms

## Abstract

**Background:**

Biological research is generating high volumes of data distributed across various sources. The inconsistent naming of proteins and their encoding genes brings great challenges to protein data integration: proteins and their coding genes usually have multiple related names and notations, which are difficult to match absolutely; the nomenclature of genes and proteins is complex and varies from species to species; some less studied species have no nomenclature of genes and proteins; The annotation of the same protein/gene varies greatly in different databases. In summary, a comprehensive set of protein/gene synonyms is necessary for relevant studies.

**Results:**

In this study, we propose an approach for protein and its encoding gene synonym integration based on protein ontology. The workflow of protein and gene synonym integration is composed of three modules: data acquisition, entity and attribute alignment, attribute integration and deduplication. Finally, the integrated synonym set of proteins and their coding genes contains over 128.59 million terminologies covering 560,275 proteins/genes and 13,781 species. As the semantic basis, the comprehensive synonym set was used to develop a data platform to provide one-stop data retrieval without considering the diversity of protein nomenclature and species.

**Conclusion:**

The synonym set constructed here can serve as an important resource for biological named entity identification, text mining and information retrieval without name ambiguity, especially synonyms associated with well-defined species categories can help to study the evolutionary relationships between species at the molecular level. More importantly, the comprehensive synonyms set is the semantic basis for our subsequent studies on Protein–protein Interaction (PPI) knowledge graph.

**Supplementary Information:**

The online version contains supplementary material available at 10.1186/s12859-023-05464-0.

## Introduction

Most protein databases have provided rich data and free access. However, each individual database has its own specific scope and can only shed light on a limited set of scientific questions. Linking protein information from multiple data sources can thus inspire new insights and offer studies over a bigger picture. However, massive data sources often have complex interrelationships and heterogeneous syntax, semantics, and schema. There are some obstacles for integration of proteins and their encoding genes information as follows.

### The names of proteins and their encoding genes are difficult to match with each other

The processes of discovery of proteins, coding genes, and these proteins being understood physiologically do not always occur simultaneously, so they do not always match each other. Scientists tend to favor one notation or name system for proteins and another for genes. Therefore, the names of genes and proteins may have their own terminologies that are always unrelated.

### Proteins and their encoding genes are often associated with multiple names and notations

For example, synonyms of the gene PPP2R3B_HUMAN are as many as six synonyms in different databases (Table1). Nomenclature sustainable revision brings enormous synonyms for protein and gene. Take the case of protein "PUR9", which is commonly referred to as "PURH", "IMPCHASE", "EL-S-70P", "AICARFT", and "ATIC" for HUMAN, but it only has one name "tapurH" for JANSC (a genus of Rhodobacteraceae). Some of the earlier names may be deprecated in favor of newer ones, although such deprecation is voluntary. Some earlier names and notations remain, as they have been widely used in the scientific literature and well established among users.

**Table 1:** Synonyms of PPP2R3B_HUMAN

Data base	Synonyms of PPP2R3B_HUMAN
UniProt	PPP2R3 L
BioGRID	PPP2R3 L; PR48; NYREN8; PPP2R3LY; LL0YNC03-56G10.1
NCBI Gene	PPP2R3 L; PR48; NYREN8; PPP2R3LY; PR70

	Synonyms of ZNT10_HUMAN
UniProt	SLC30A10; ZNT10; ZNT8; Zinc transporter 10
BioGRID	SLC30A10; ZNT10; ZNT8; HMDPC; ZRC1; ZnT-10
NCBI Gene	SLC30A10; ZNT10; ZNT8; HMDPC; ZRC1; ZnT-10; HMNDYT1

### There is no gene and protein nomenclature for less popular species

At present, guidelines for nomenclature of proteins and genes in mice, rats, chickens, humans, nonhuman primates, domestic animals, flies (fruit flies) and fish have been published and subsequently revised since 1940 [1–4]. However, there is no consensus among biologists about the nomenclature of other species to date. In this situation, arbitrary naming of those genes and proteins poses a major challenge for data integration.

### The nomenclature for genes and proteins is complex and varies with species

To distinguish species with the same proteins and genes in different organisms, nomenclatural systems provide human-versus-nonhuman specificity by using different capitalization, such as the name "PURH" in species of HUMAN and "purH" in species of JANSC. However, scientists often ignore the specification. As a result, there are various synonyms for identical proteins and genes among different species.

### Comprehensive protein/gene synonyms are a must

Protein databases have recorded a huge number of protein synonyms. SwissProt records 1,098,808 synonyms, NCBI Gene has accumulated 218,622 synonyms and BioGRID has 139,587 synonyms (Table 2). None of them can collect all of the protein and its encoding gene synonyms. For example, the synonyms of the gene PPP2R3B_HUMAN are stored in different databases that not only overlap but also differ, as shown in Table 1. Therefore, comprehensive integration of these data is very necessary for research in related fields.

**Table 2:** Data source information

Data source (modified-date)	Synonyms number	Entities number (size)	Entity types	Characteristics
BioGRID (27-Aug-2019)	1,098,808	104,563 (6.46G)	Genes, Proteins	Synonyms are mostly involved in the literatures describing protein interactions
UniProtKB/Swiss-Prot (8-May-2019)	139,587	560,275 (6.26G)	Proteins, Protein sequences	Focusing on mining and recording synonyms related to sequences in protein literatures, while paying less attention to synonyms related to protein function and other aspects
NCBI Gene (9-Sep-2020)	218,622	25,927,333 (3.29G)	Genes	Possible synonyms for each gene

Ontology is a formal, explicit specification of a shared conceptualization [5]. At the heart of any ontology is a set of entities, also called classes, which are arranged into a hierarchy from the general to the specific. These entities that are contained in ontologies are then available for use as hubs around which data can be organized, indexed, aggregated and interpreted across multiple different services, databases, and applications [6]. In this study, we built protein and gene synonyms based on protein Ontology. Finally, a knowledge sharing platform is developed on the basis of the synonym resources and the knowledge graph.

## Methods

**Fig. 1:**
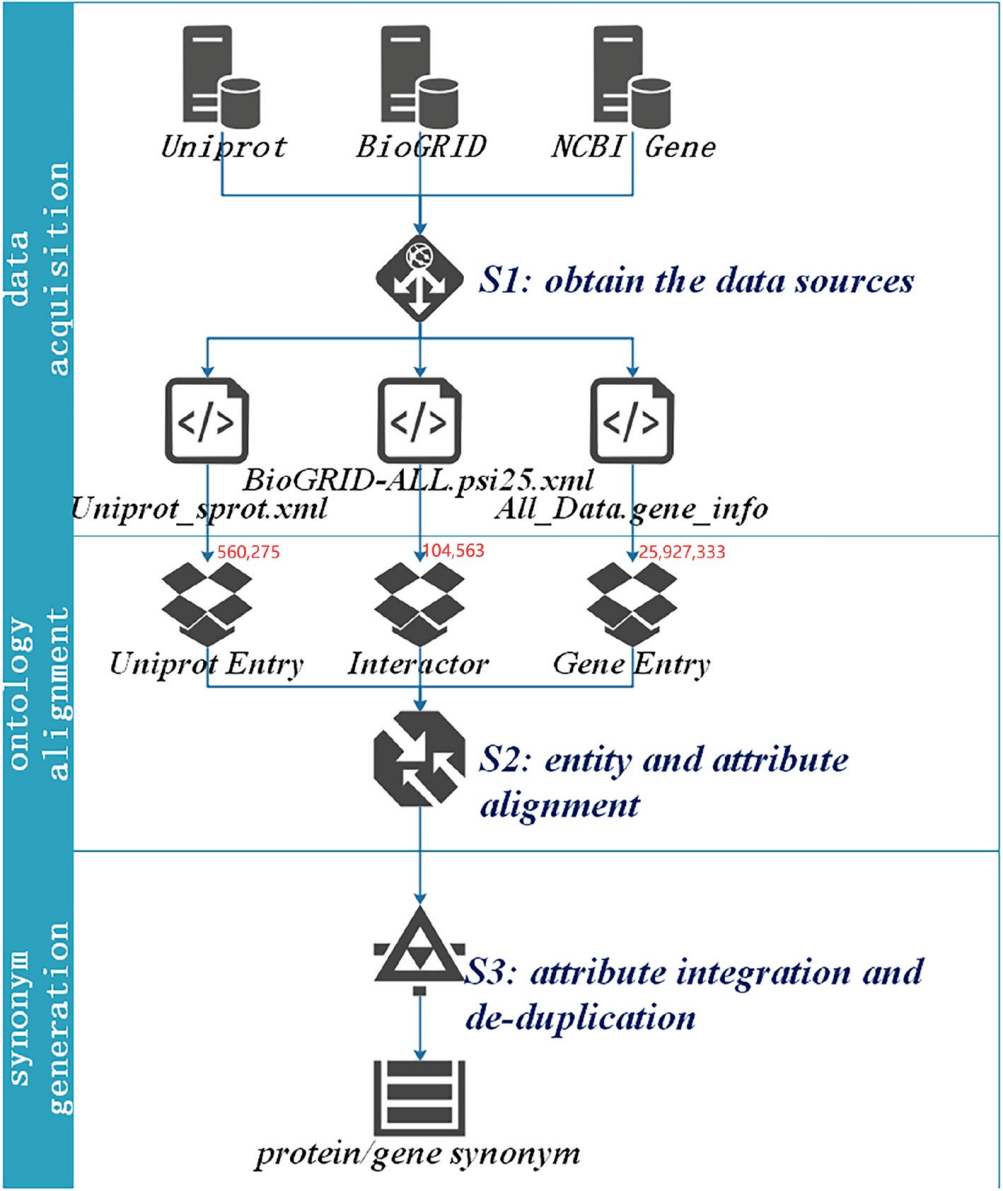
Workflow of generation for protein and gene synonym. The red font denotes entities number used for integration in each database

The workflow of protein and gene synonym integration is composed of three modules: data acquisition, entity and attribute alignment, attribute integration and deduplication, as shown in Fig. 1.

### Data collection

We selected UniProtKB, BioGrid, and NCBI Gene as the data sources for proteins and their encoding genes synonyms. UniProtKB (UniProt Knowledge base) is a public database for protein sequence and function, covering the tree of life and over 220 million protein entries. UniProtKB is a set of expert-verified data that consists of two parts: UniProtKB/Swiss-Prot and UniProtKB/TrEMBL. While UniProtKB/TrEMBL main contains unreviewed automatic annotation and classification, calculated data, UniProtKB/Swiss-Prot is now the reviewed section, containing a high quality manually annotated and non-redundant protein sequence data, which brings together experimental results, computed features and scientific conclusions. Therefore, we chose UniProtKB/Swiss-Prot for this experiment. In the version released in June 2019, UniProtKB/SwissProt contained 560,275 protein entities and is a high-quality annotated and nonredundant protein sequence database [7]. BioGRID is an open access database dedicated to the curation and archival storage of protein, genetic and chemical interactions for all major model organism species and human [8]. BioGRID-3.5.176 contains 104,563 entities [8]. NCBI Gene provides gene sequence annotation and retrieval services, NCBI Map Viewer, Evidence Viewer, Model Maker, and BLAST Link (Blink). Protein domains from the Conserved Domain will also be linked database (CDD) and other gene-related resources [9]. To preserve as much semantic information as possible, we obtained datasets in XML format from UniProtKB, BioGrid, and NCBI Gene respectively (Table 2).

As shown in Table 1, in the repository of UniProt, BioGRID and NCBI Gene, there are not only overlaps but also disparate synonyms for the same gene, such as ZNT10_HUMAN and PPP2R3B_HUMAN. As it turns out, it is hard for a specific proteomics and genomics database to provide comprehensive synonyms.

### Data integration

**Fig. 2:**
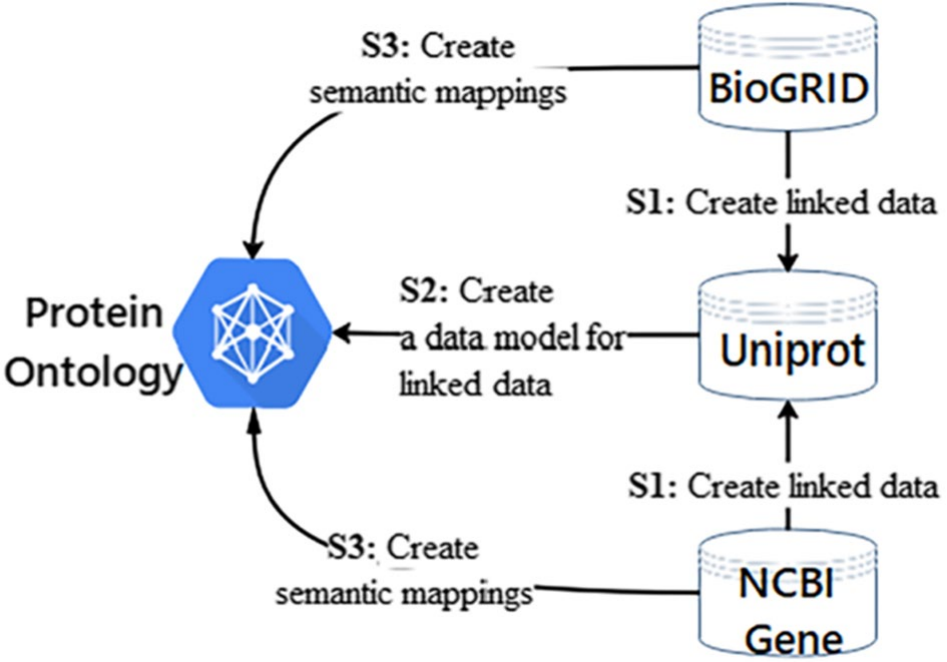
Workflow for protein and its encoding synonyms integration. A data model is the abstract definition of data that describes its structure, data semantics, relationships, and data constraints

We proposed an ontology-based method to integrate species-dependent synonyms. First, we created links for protein data based on external links from BioGRID to UniProt. Second, we created links for protein-encoding genes based on external links from NCBI Gene to UniProt according to “GeneID”. Third, protein ontology was built as a data schema and semantic specification. Finally, the synonyms of proteins and their encoding genes were integrated based on protein ontology and species information. The workflow is shown in Fig. 2.

### S1 creating linked data based on cross-referencing

**Fig. 3:**
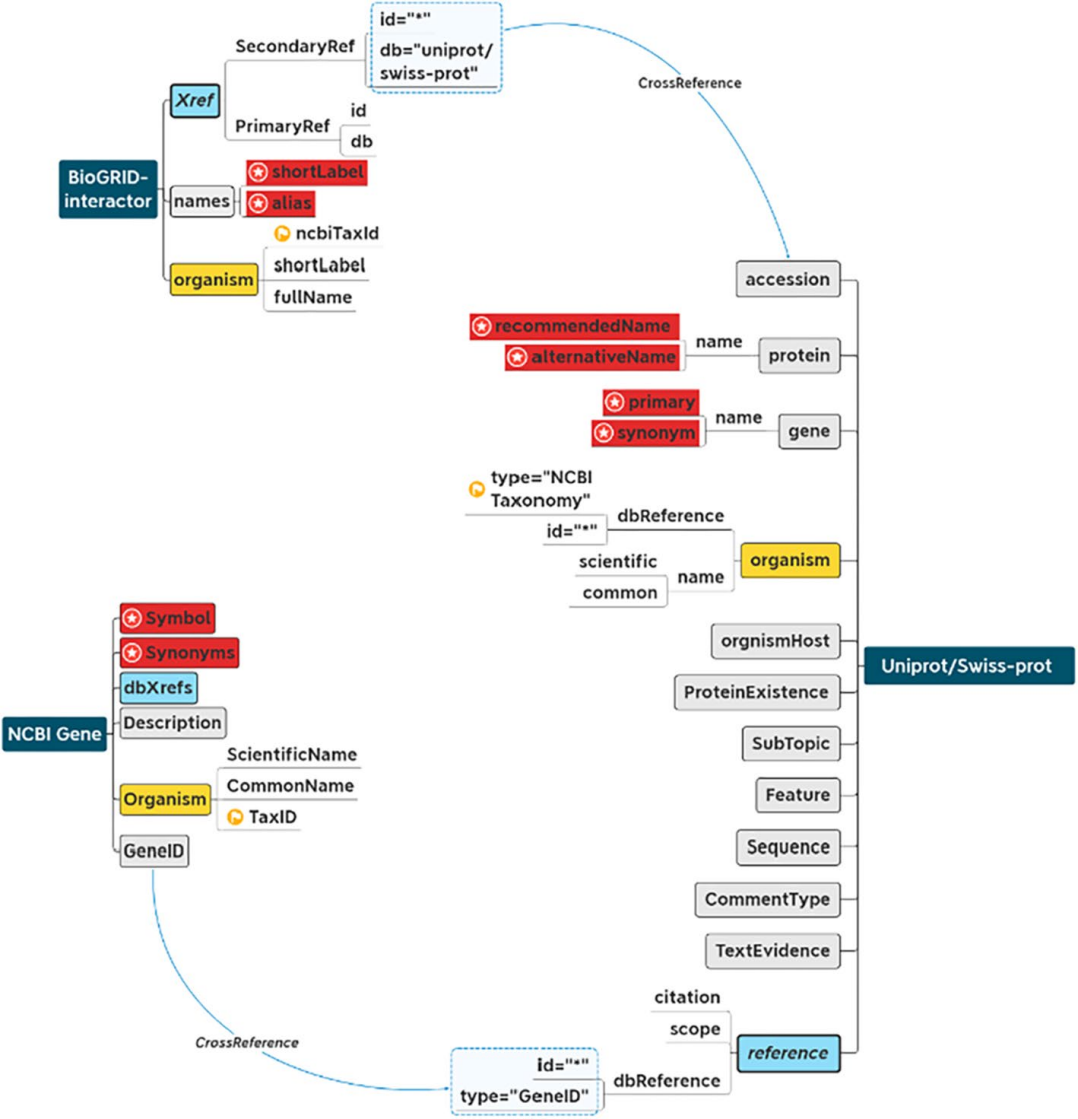
Create linked data rely on cross-reference. The blue tags denote integration of cross-references, the red tags denote integration of names and synonyms of protein/genes, and the yellow tags denote integration of species taxonomy information. The blue lines indicate cross-reference relationships between databases

Tags of CrossReference, Synonyms and Taxonomy were recorded in different labels as shown in Table 3. Tags of the BioGRID interactor, UniProt/SwissProt, and NCBI Gene were extracted as shown in Fig. 3, then their cross-references were ecorded in <Xref>, <dbXrefs> and <reference >, respectively. Cross-references were marked with light blue tags (Fig. 3). Protein identifiers were recorded in <id > and <accession > of BioGRID and UniProt/SwissProt, respectively. The gene identifier was recorded in <Gene ID > NCBI Gene (Fig. 3). These tags were used for data integration. Proteins and their encoding genes were linked through protein identifiers and gene identifiers, respectively, as shown by the light blue lines in Fig. 3.

**Table 3:** The synonyms within linked data

DataSource	CrossRef labels	Synonyms labels	Taxonomy labels
BioGRID	<Xref>	<shortLabel>, <alias>	<ncbiTaxId>
NCBIGene	<dbXrefs>	<Symbol>, <Synonyms>	<TaxID>
UniProt/swiss-prot	<reference>	<recommendedName>, <alternativeName>, <primary>, <synonym>	<type="NCBI Taxonomy">

### S2 creating a protein ontology schema based on the UniProt Knowledge Base

**Fig. 4:**
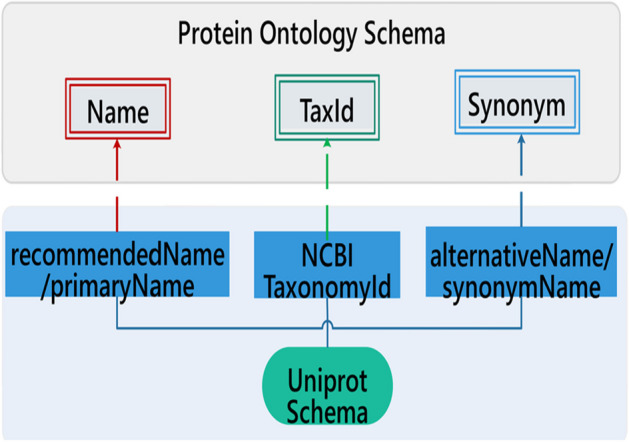
Schema of protein ontology

The schema of the UniProt Knowledge Base was extracted as basis of the protein ontology schema, as shown in Fig. 4. The classes of “Name”, “TaxId”, and “Synonym” were created for concepts of genes’ and proteins’ primary names, species id, and synonyms of genes and proteins, respectively.

### S3 creating semantic mappings

First, we converted all the names to lowercase. Second, semantic mappings were created as follows:

#### Semantic mappings between UniProt and protein ontology

As shown in Fig. 4, UniProt tags <recommendedName >, <PrimaryName > were mapped to ‘Name’ in Protein Ontology. Tags <alternativeName > and <synonymName > were mapped to ‘Synonym’ in Protein Ontology. Tag <NCBI TaxonomyId > was mapped to ‘TaxId’ in Protein Ontology. Meanwhile, values of tag <Synonym > were saved as synonyms of proteins automatically. Here, the value of tag refers to content between two tags in an HTML file. For example, in <TaxID > 8355 </TaxID >, 8355 is the value of <TaxID >. Proteins and associated properties were saved as instances of protein ontology.

#### Semantic mappings between BioGRID and protein ontology

**Fig. 5:**
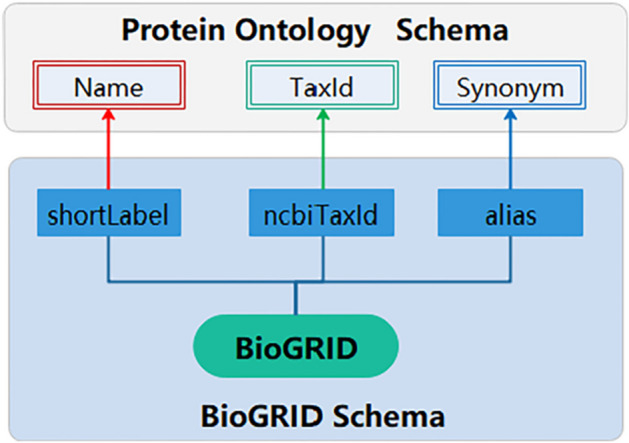
Semantic mappings between BioGRID and Protein Ontology

As shown in Fig. 5, BioGRID tags <shortLabel >, <ncbiTaxId > and <alias > were mapped to Protein Ontology class ‘Name’, ‘TaxId’, and ‘Synonym’, respectively. As the semantic mappings were created, data in the BioGRID tags can be integrated automatically subsequently. Meanwhile, proteins and related attributes were saved as instances of protein ontology, and values of tag <alias> were saved as synonyms of proteins.

#### Semantic mappings between NCBI Gene and protein ontology

**Fig. 6:**
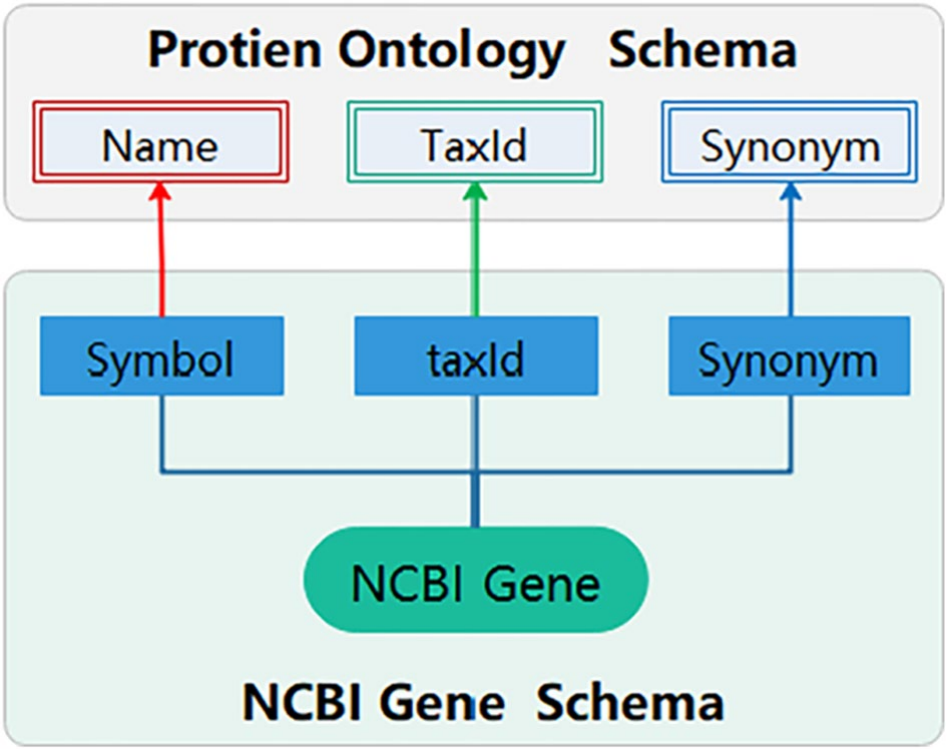
Semantic mappings based on protein ontology

As shown in Fig. 6, tags <Symbol >, <taxId >, and <Synonym > were mapped to the Protein Ontology class ‘Name’, ‘TaxId’, and ‘Synonym’, respectively. Data in NCBI Gene tags were saved as instances of protein ontology, and values of tag <Synonym > were saved as synonyms of the gene automatically.

### Synonym integration and classification

**Fig. 7:**
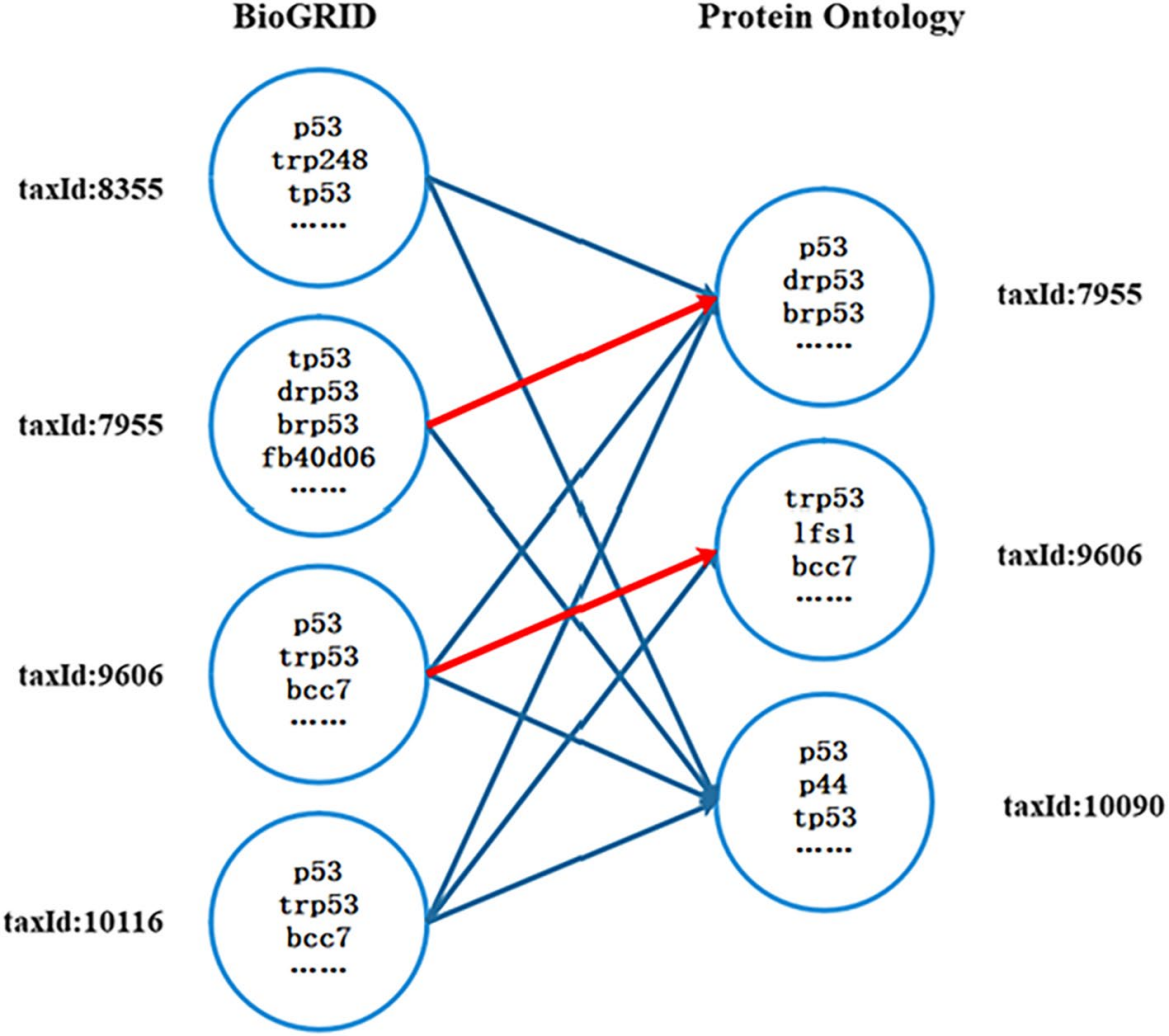
Synonyms matching rely on species information

Names of proteins and their encoding genes were obtained based on semantic mappings as related in above section. As shown in Fig. 7, there were a multitude of many-to-many protein name mappings between BioGRID and Protein Ontology that rely on protein ID. Approximate 70% many-to-many name mappings were incorrect and were caused by different species. Since most many-to-many error mappings were caused by species differences, we checked the mappings with the species ID again. The name mappings with inconsistent taxId denoted by blue lines were ignored. The rest of the mappings with the same taxId denoted in red lines were saved.

## Results and discussion

**Fig. 8:**
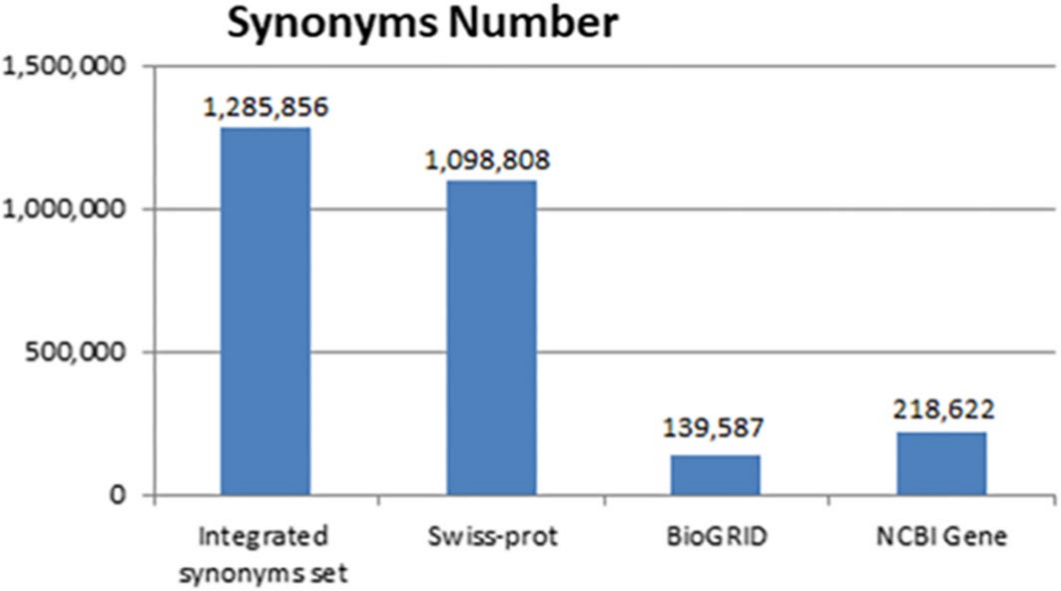
The total number of synonyms in different database

In this study, we proposed an approach for proteins and their encoding genes synonym integration based on protein ontology. The Protein ontology was developed based on Uniprot Knowledge Base. Rely on protein ontology, 560,275 proteins and coding genes from BioGrid, Uniprot and NCBI Gene databases were linked together. As a result, there were 128.59 million proteins and their coding genes’ terminologies in the integrated synonym set, which was much higher than the number of synonyms in each database (Fig. 8).

Using the comprehensive integrated synonyms as a dataset, we developed a Protein–protein interaction (PPI) knowledge graph and a data platform [10] to provide one-stop data retrieval without considering the diversity of protein nomenclature and species. Here, the synonyms set played the role of semantic mediative middleware in the PPI knowledge graph, and are also the semantic support of the data platform (Refer to Additional file 1 for website instructions).

**Fig. 9:**
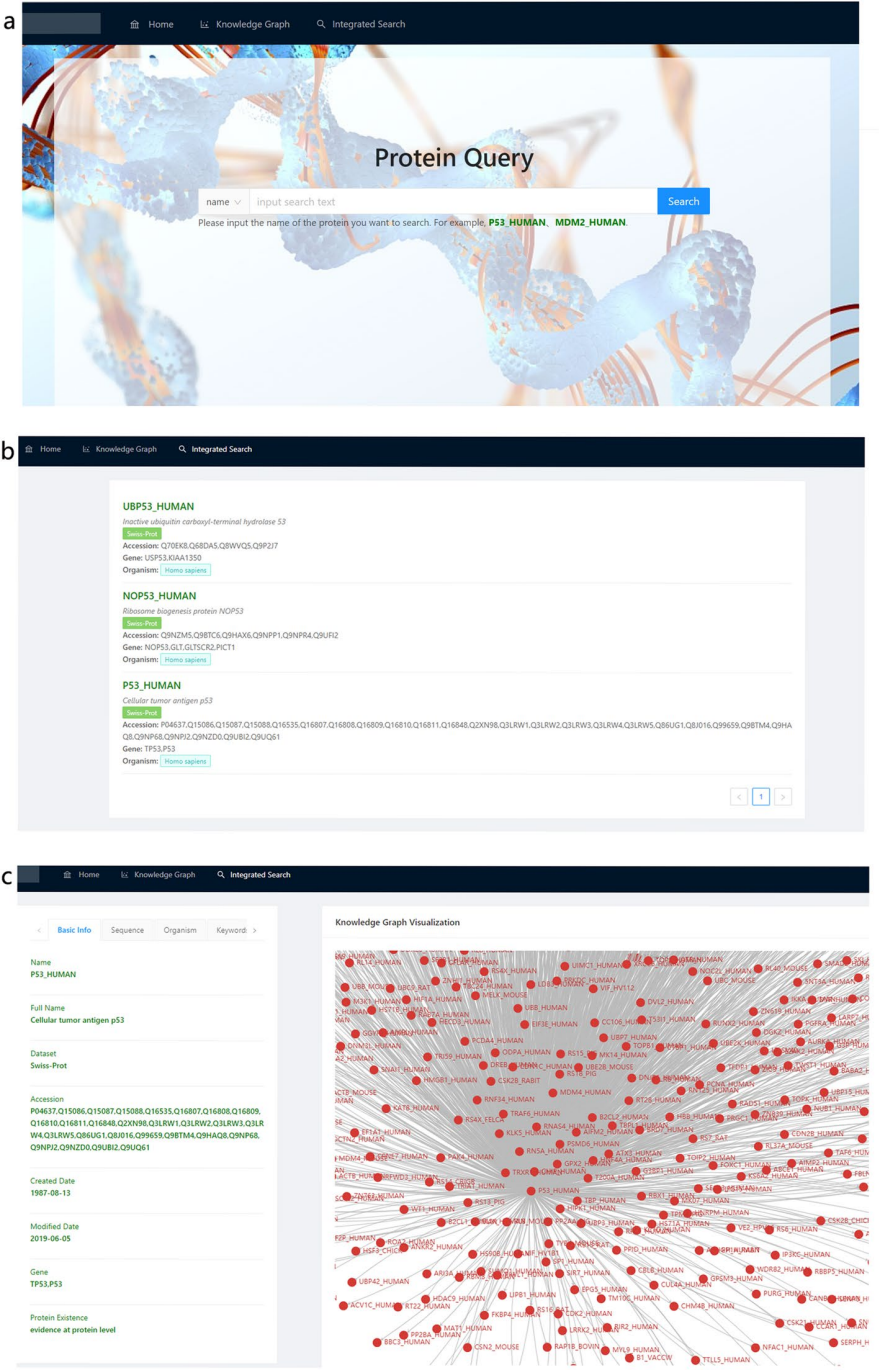
PPI Knowledge Graph of P53_HUMAN. **a** query page; **b** results of P53_HUMAN protein query based on synonym set; **c** the details page

On the protein query page [10], users can get comprehensive and integrated protein information in one-stop, no matter which name of a species protein is entered, without going to Uniprot, NCBI Gene, or BioGRID databases one by one. P53-HUMAN is widely studied and named many times, take the protein as an example, with the support of synonyms set, users can input a random noun to get the knowledge graph of P53_HUMAN and its related proteins (Fig. 9a). When the users query P53_HUMAN, its synonyms and primary information is listed on the page, as shown in Fig. 9b. A detailed page consisting of “Basic Info”, “Sequence”, etc. is displayed on the left side of the page (Fig. 9c), and its PPI knowledge graph is shown on the right side.

**Fig. 10:**
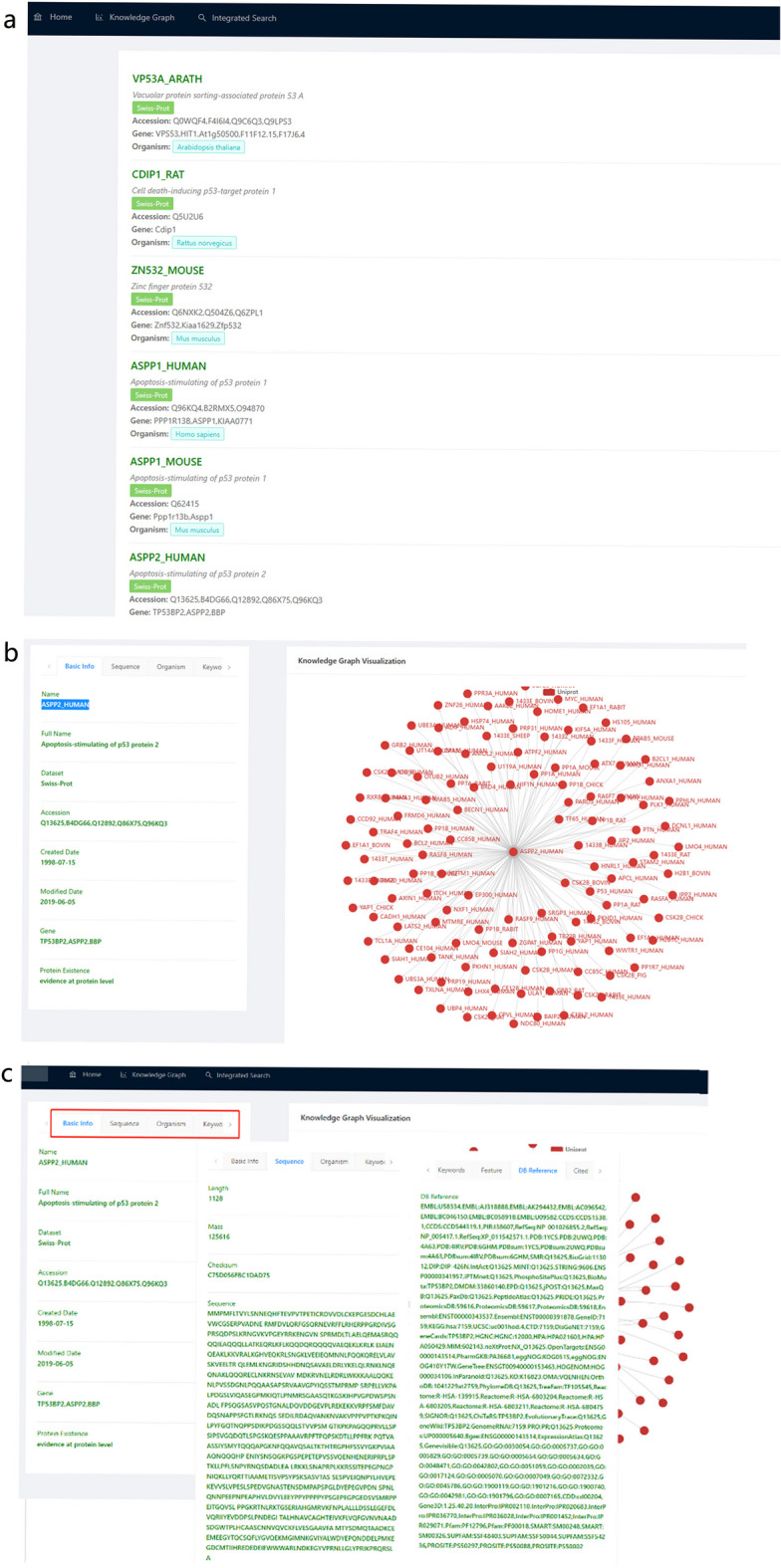
PPI Knowledge Graph of P53 protein (partially displayed). **a** search results of P53 protein in different species; **b** ASPP2_HUMAN tabs; **c** details page of a tab

By identifying the species information recorded in the organism tags in the three data sources, we in turn assigned species classification information to these term sets, which statistically cover 13,781 species. As a result, each protein and its coding gene terminology is greatly integrated on the basis of species information with attribute information such as sequence, scientific name, accession, related gene, organism and data origin. When user searches for P53 in the data platform [10], the protein data of all species in the three data sources can be obtained (Fig. 10a). When a user clicks on a protein (take ASPP2_HUMAN for example), there are 7 tabs of basic info, sequence, organism, keywords, feature, DB reference, cited on the left side; the knowledge graph of PPI centered on it can be seen on the right side (Fig. 10b). There are 7 tabs covering sequence, scientific name, accession, related gene, organism, data origin and other information comprehensively (Fig. 10c). Such as basic info option card covers the Name, Full Name, the Dataset, Accession, Created the Data, the Modified Data, Gene, Protein Existence. Protein sequence option card shows the Length, Mass, Checksum, Sequence data. It is worth mentioning that users can click any protein in PPI knowledge graph on the right to obtain the above comprehensive information, avoiding multiple queries to multiple databases.

In brief, this synonyms set with species taxonomic information can not only map different protein names to the same protein scientific name, but also cluster the protein names of the same species according to the species taxonomic information. This work is fundamental to the alignment of proteins and their coding gene entities in the development of the PPI knowledge graph, and is an indispensable backbone for the subsequent semantic and species-based retrieval of proteins and their interaction information.

## Conclusion and future work

In summary, the data integration approach based on protein ontology can provide more comprehensive synonyms for proteins and their coding genes. Through a three-level workflow of data collection, entity and attribute alignment, attribute integration and duplicate data removal, each protein and its coding gene term was greatly integrated with attribute information such as sequence, scientific name, accession, related gene, organism and data source on the basis of species information. 1. The final constructed synonym set can be used as an important resource for biological named entity identification, text mining and information retrieval without name ambiguity, especially synonyms associated with well-defined species categories can help to study the evolutionary relationships among species at the molecular level. It is of great significance for comprehensive acquisition of species taxonomic information and study of organism’s evolutionary history by means of inheritance and homology of common lineages, as well as the conserved nature of sequence and sequence structure that determine function.

2. More importantly, the comprehensive synonyms set is the semantic basis for our subsequent studies on PPI knowledge graph. It offers a retrieval tool which can easily get protein information based on PPI knowledge graph without consideration protein arbitrary naming and lacking species membership information.

In the future, we will provide Application Programming Interfaces (APIs) for free to allow more terminologies to be integrated to sustainably enhance the size and scalability of proteins and their encoding gene synonyms.

### Supplementary Information

**Additional file 1:** Protein Query Website Instructions.

## Data Availability

The datasets used for this study are available online at https://downloads.thebiogrid.org/File/BioGRID/Release-Archive/BIOGRID-3.5.175/BIOGRID-ALL-3.5.175.psi25.zip, Index of /pub/databases/uniprot/previous_releases/release-2019_05/knowledgebase and https://ftp.ncbi.nih.gov/gene/DATA/. All data generated during this study are available upon reasonable request from the corresponding author.
