# Supplementary material for: An approach for proteins and their encoding genes synonyms integration based on protein ontology
Source: BMC Bioinformatics. 2023 Sep 12;24:339. doi: 10.1186/s12859-023-05464-0 (PMC10496362; doi:10.1186/s12859-023-05464-0)
Supplement: Supplementary file 1 — Additional file 1: Protein Query Website Instructions. [file 12859_2023_5464_MOESM1_ESM.pdf]

Protein Query Website Instructions

1. Function Introduction

The protein query website (http://43.153.4.4:8000/protein_search) constructed on the currently integrated synonym of proteins and their encoding genes. It can provide users with a one-stop query containing clear species classification information for each protein, covering sequence, scientific name, accession, related gene, organism, data origin and other information comprehensively. In addition, by embedding the protein interaction knowledge graph, users can obtain protein-protein interaction information associated with the retrieved protein simultaneously.

In the future, we will sustainable update the protein synonym data incrementally to improve the recall of the query. Next, we plan to add modules that demonstrate the three-dimensional structure of proteins.

2. Instructions and examples

2.1 Protein Search

A Protein Query page is shown as Figure S1. Search bar is designed for inputting keywords of the protein. Keywords could be protein name, accession, sequence and etc. After selecting the type of keyword on the left side of the search bar, user types the corresponding content on the right side. For example, after selecting "keyword", type "Disease mutation" into the text box.

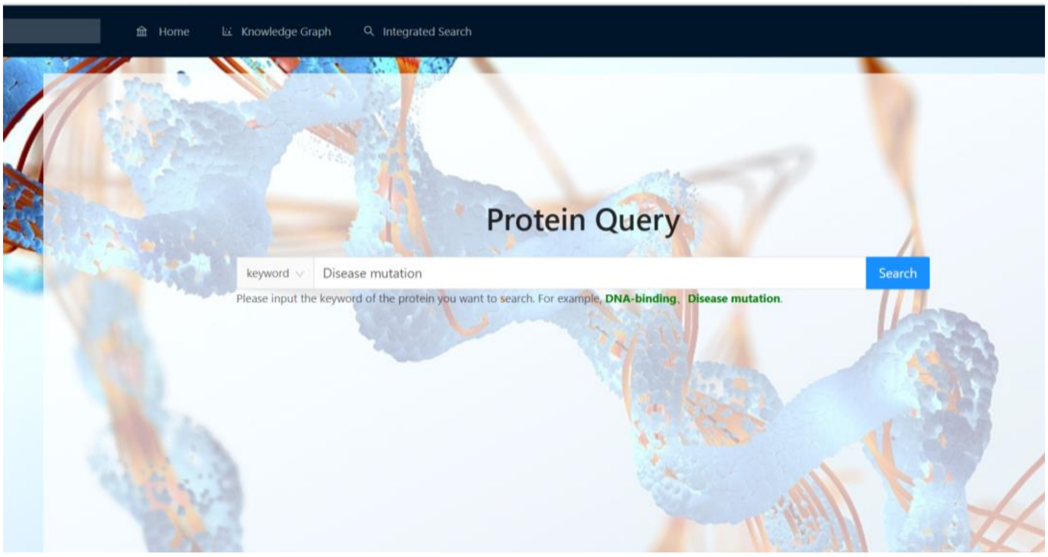

Figure S1 Protein Query Page

2.2 Results presentation

After clicking on the SEARCH button, the page will go to the results page. As shown in Figure S2, the query results are based on the protein synonym and there are 30 pages (10 entries each page) of protein data entries related to the keyword “Disease mutation”; each record shows protein names (in green font) and related information such as Accession, Gene, and Organism. When clicking on one of the proteins, the page will jump to the detail page of the protein. For example, when clicking on "SAHH_HUMAN", the page will go to the detail page (Figure S3).

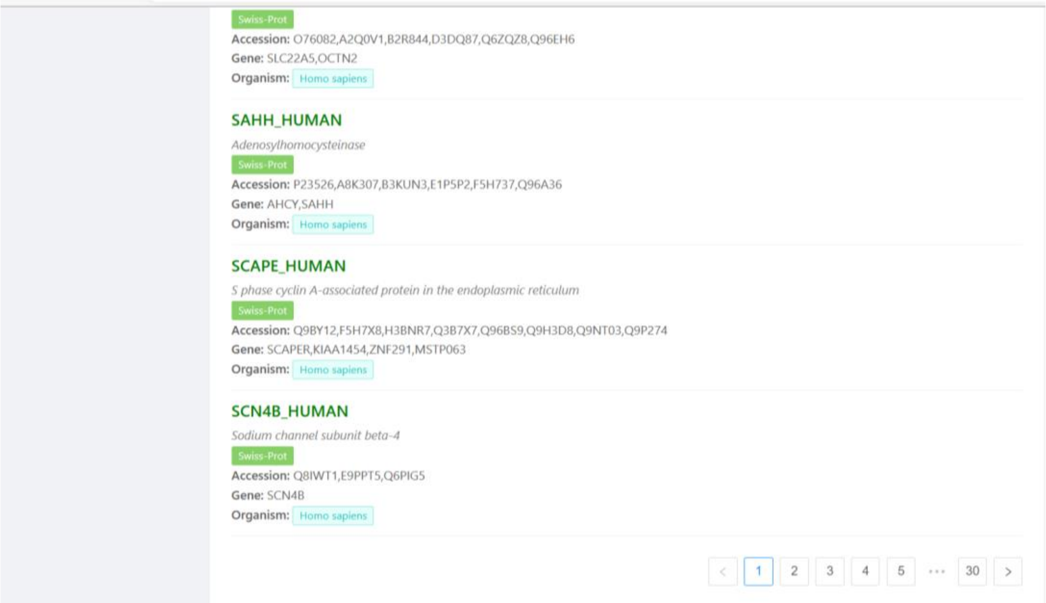

Figure S2 Results based on protein synonym

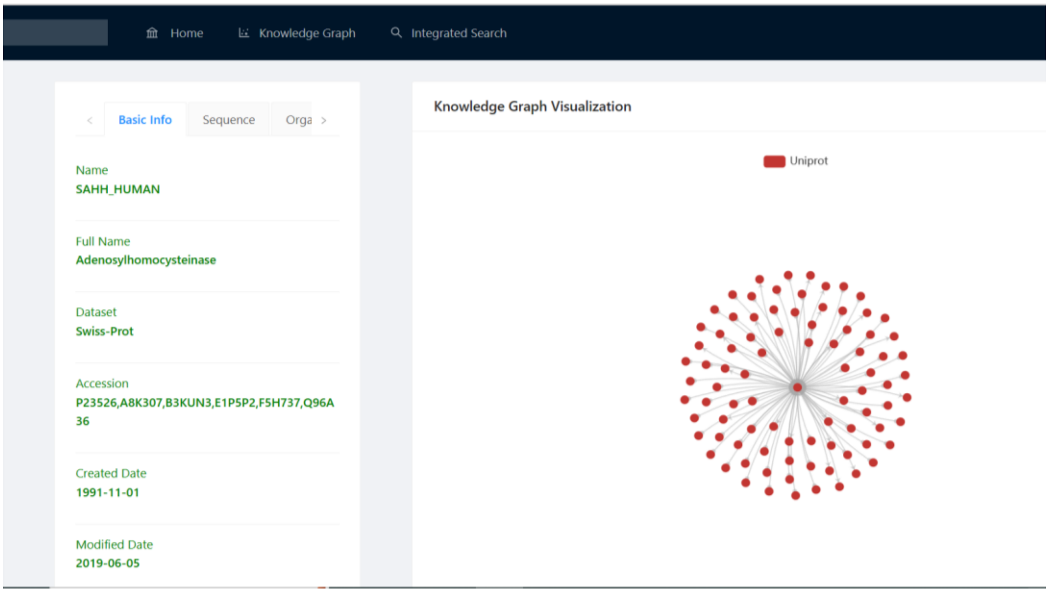

Figure S3 ”SAHH_HUMAN” detail page

There are 7 tabs: Basic Info, Sequence, Organism, Keywords, Feature, DB Reference and Cited (at the top-left corner in Figure S3). Clicking on ‘Basic info’ option card, it will display: Name, Full Name, Dataset, Accession, Created date, Modified date, Gene, Protein Existence (Figure S3). “Sequence” option card can check the Length, Mass, Checksum and Sequence data (Figure S4).

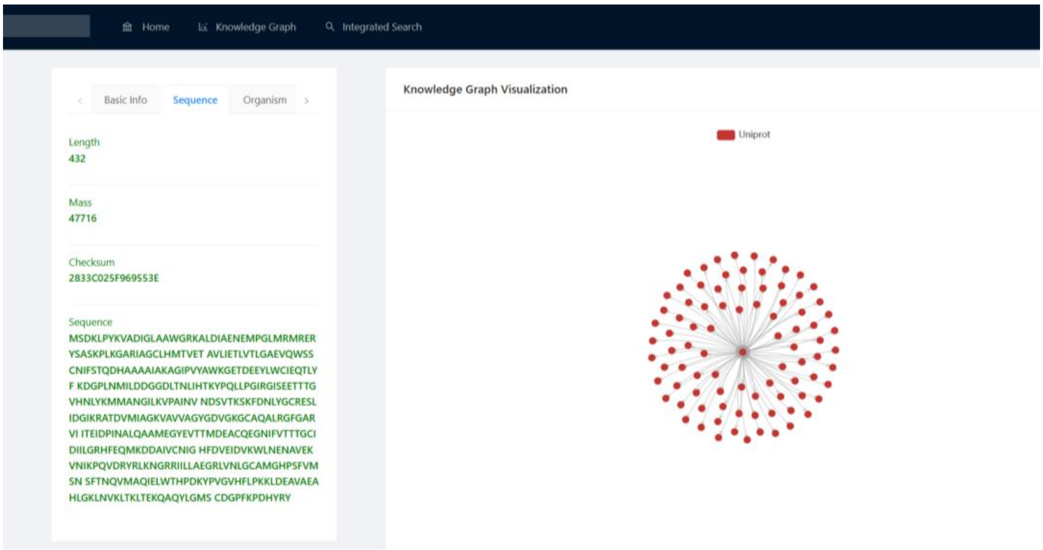

Figure S4 Sequence information in “Sequence” option card

2.3 Access to the PPI knowledge graph

In the PPI knowledge graph (PPIKG) on the right side of the page, user can learn which proteins interact with “SAHH_HUMAN” and can obtain the information about it with one click (Figure S5). For example, if you click protein “CUL1_HUMAN” in PPIKG, the detail information about it will be displayed in the Entity Detail card on the right side (Figure S6).

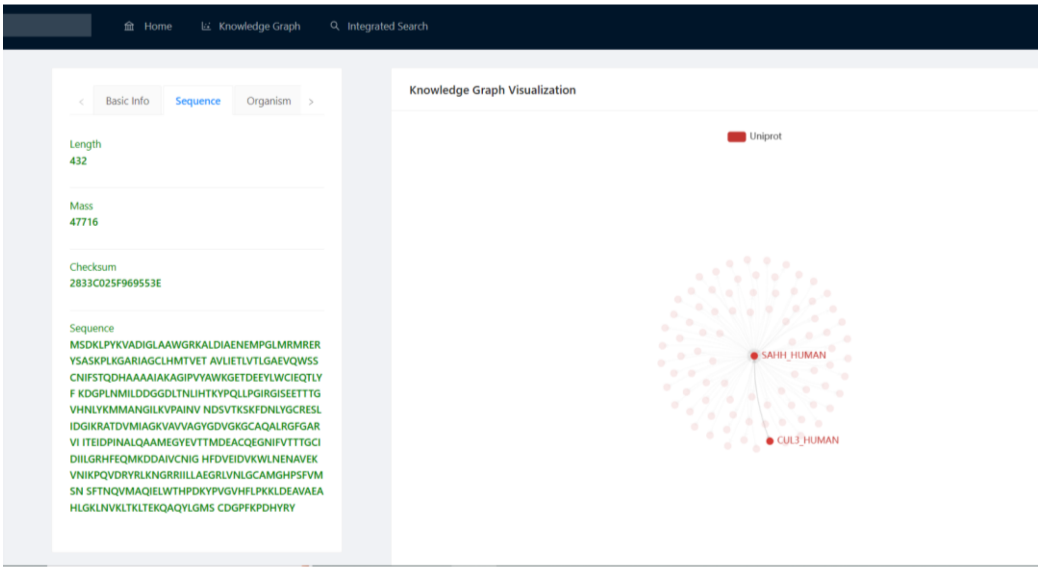

Figure S5 PPI KG page

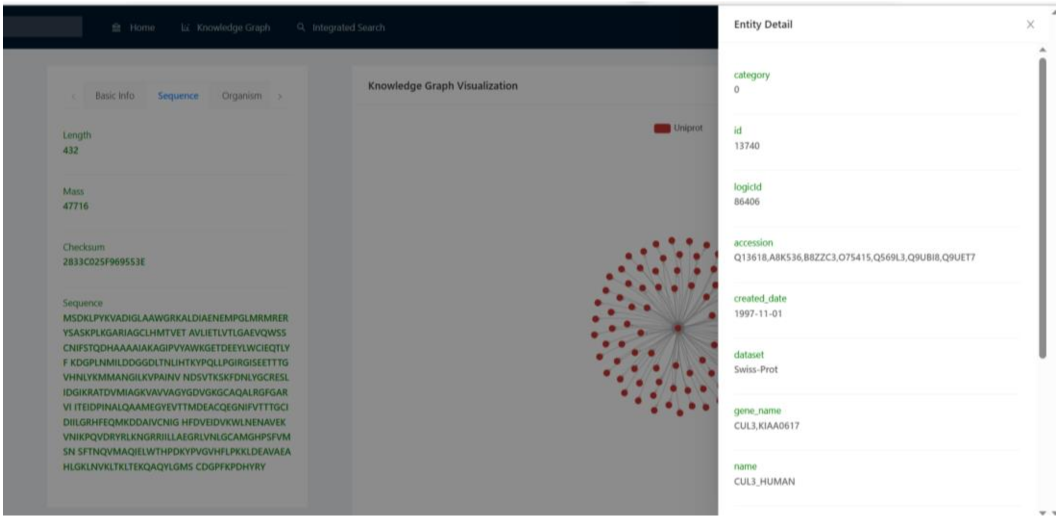

Figure S6 Detail page of "CUL1_HUMAN"

2.4 Download of PPI knowledge graph node data

User can download list of nodes and related information in PPI Knowledge Graph page (The PPI Knowledge Graph menu is below "Knowledge Graph" menu), and the condition query is on the left part of the page (Figure S7). After entering target protein information in the "Head Node" part, the list of nodes and the related information can be downloaded in csv format.

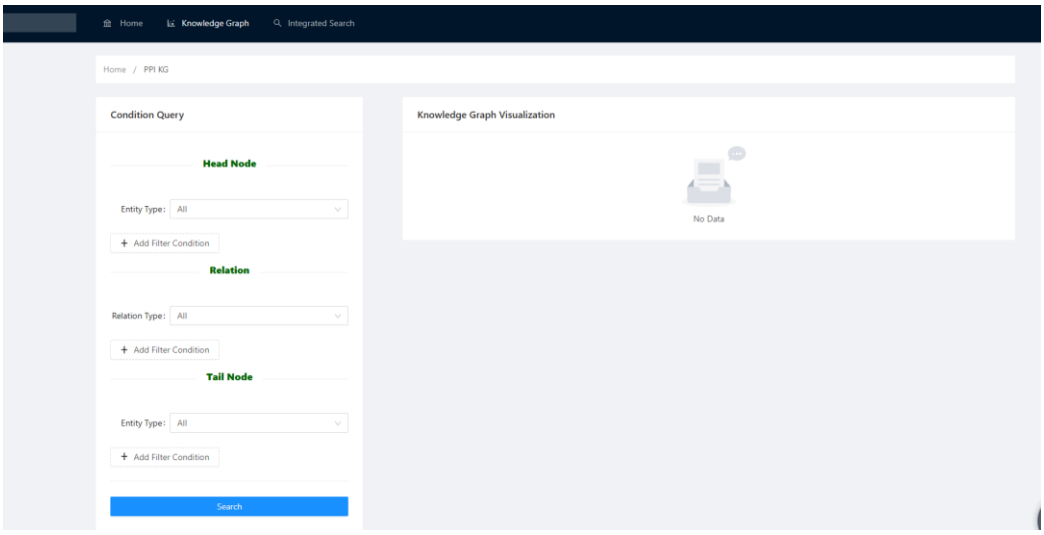

Figure S7 Knowledge Graph page

For example, if user wants to learn detail PPI knowledge graph nodes information of "CUL1_HUMAN", after clicking on the "Add Filter Condition" button (be part of "Head Node"), selecting "entity type" as Uniprot (our protein data source), "Property" as name, "Condition" as Equal To and typing "CUL1_HUMAN" into the "Value" box, then clicking on the "search" button, the download button is appeared at the top of the PPI knowledge graph (Figure S8).

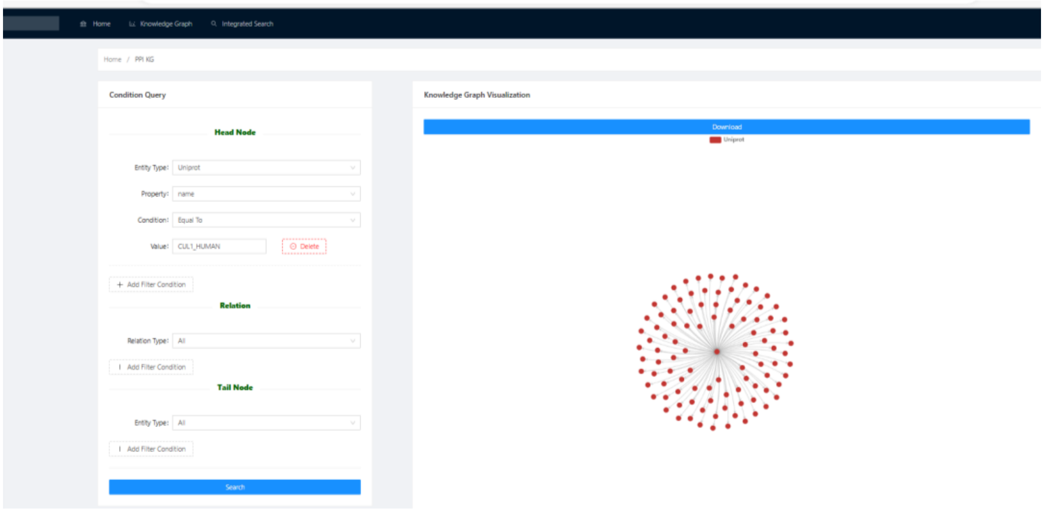

Figure S8 Download page

After clicking on the "download" button, table in cvs format of PPI knowledge graph can be saved. In the table, information about each node (protein) includes: logicId, sequence_length s, equence_mass, dataset, created_date, modified_date name, protein FullName, geneName, accession, organism_scientific_name (Figure S9).

	logicId	sequence_length	sequence_mass	dataset	created_date	modified_date	name	protein FullName	geneName	accession	organism_scientific_name
2	542922	776	89679	Swiss-Pro	1997-11-	2019-06-	CUL1_HUI	Cullin-1	CUL1	Q13616 D3	Homo sapiens
3	493344	341	37718	Swiss-Pro	2007-06-	2019-05-	S2543_HU	Solute ca	SLC25A4:	Q8WUT9 07	Homo sapiens
4	18249	245	26860	Swiss-Pro	1986-07-	2019-06-	BZLF1_EI	Trans-act	BZLF1	P03206 Q6	Epstein-Barr
5	557260	729	80272	Swiss-Pro	1998-07-	2019-06-	DDX17_HU	Probable	DDX17	Q92841 B1	Homo sapiens
6	98457	529	57862	Swiss-Pro	1996-10-	2019-06-	IMA1_HUI	Importin	KPNA2 I	P52292 B9	Homo sapiens
7	34291	312	34353	Swiss-Pro	2001-10-	2019-06-	AIMP1_HU	Aminoacyl	AIMP1 I	Q12904 B3	Homo sapiens
8	120550	105	12254	Swiss-Pro	2000-05-	2019-06-	RL36_HUI	60S ribos	RPL36	Q9Y3U8 B2	Homo sapiens
9	76954	377	42051	Swiss-Pro	1986-07-	2019-06-	ACTS_HUI	Actin a	ACTA1 /	P68133 P0	Homo sapiens
10	144949	487	53481	Swiss-Pro	2001-01-	2019-05-	BAT1_HUI	b(0 +)-t	SLC7A9	P82251 B2	Homo sapiens
11	522214	540	60674	Swiss-Pro	1997-11-	2019-06-	ECM1_HUI	Extracell	ECM1	Q16610 A8	Homo sapiens
12	127636	248	27745	Swiss-Pro	1995-02-	2019-06-	SRSF1_HU	Serine/ar	SRSF1 /	Q07955 B2	Homo sapiens
13	29688	594	68208	Swiss-Pro	2000-12-	2019-06-	GLMN_HUI	Glomulin	GLMN F/	Q92990 Q5	Homo sapiens
14	362600	208	24205	Swiss-Pro	2004-07-	2019-06-	RS8_MOUS	40S ribos	Rps8	P62242 P0	Mus musculus
15	203337	353	41401	Swiss-Pro	2003-05-	2019-06-	BRX1_HUI	Ribosome	BRIX1 I	Q8TDN6 A8	Homo sapiens
16	130560	444	49671	Swiss-Pro	1987-08-	2019-06-	TBB5_MOU	Tubulin b	Tubb5	P99024 B1	Mus musculus
17	460254	474	54235	Swiss-Pro	1990-11-	2019-06-	GBRB1_HU	Gamma-ami	GABRB1	P18505 B2	Homo sapiens
18	182986	133	15423	Swiss-Pro	2004-08-	2019-06-	RS24_HUI	40S ribos	RPS24	P62847 E7	Homo sapiens
19	154390	286	30633	Swiss-Pro	2007-05-	2019-06-	FBSP1_HU	F-box/SPI	FBXO45	P0C2W1 A6	Homo sapiens
20	20482	1640	187030	Swiss-Pro	1996-10-	2019-05-	CLH2_HUI	Clathrin	CLTCL1	P53675 B7	Homo sapiens
21	308436	448	49924	Swiss-Pro	1987-08-	2019-05-	TBA4A_MO	Tubulin a	Tuba4a	P68368 P0	Mus musculus
22	491928	263	29598	Swiss-Pro	2004-07-	2019-06-	RS4X_HUI	40S ribos	RPS4X (	P62701 P1	Homo sapiens
23	296003	211	24261	Swiss-Pro	1992-08-	2019-06-	RL13_HUI	60S ribos	RPL13 I	P26373 B4	Homo sapiens

Figure S9 Part of CUL1_HUMAN' knowledge graph nodes information table
